# Immune checkpoint receptors: homeostatic regulators of immunity

**DOI:** 10.1007/s12072-018-9867-9

**Published:** 2018-05-08

**Authors:** Antonio Riva, Shilpa Chokshi

**Affiliations:** 1 Institute of Hepatology London, Foundation for Liver Research, 111 Coldharbour Lane, London, SE5 9NT UK; 2 Faculty of Life Sciences and Medicine, King’s College London, London, UK

**Keywords:** Checkpoint, Immunotherapy, ALD

## Abstract

Alcoholic liver disease (ALD) is an escalating global problem accounting for more than 3 million deaths annually. Bacterial infections are diagnosed in 25–47% of hospitalized patients with cirrhosis and represent the most important trigger for acute decompensation, multi-organ failure, septic shock and death. Current guidelines recommend intensive antibiotic therapy, but this has led to the emergence of multi-drug resistant bacteria, which are associated with increased morbidity and mortality rates. As such, there is a pressing need to explore new paradigms for anti-infective therapy and host-directed immunomodulatory therapies are a promising approach. Paradoxically, cirrhotic patients are characterised by heightened immune activity and exacerbated inflammatory processes but are unable to contend with bacterial infection, demonstrating that whilst immune effector cells are primed, their antibacterial effector functions are switched-off, reflecting a skewed homeostatic balance between anti-pathogen immunity and host-induced immunopathology. Preservation of this equilibrium physiologically is maintained by multiple immune-regulatory checkpoints and these feedback receptors serve as pivotal regulators of the host immunity. Checkpoint receptor blockade is proving to be effective at rescuing deranged/exhausted immunity in pre-clinical studies for chronic viral infection and sepsis. This approach has also obtained FDA approval for restoring anti-tumor immunity, with improved response rates and good safety profiles. To date, no clinical studies have investigated checkpoint blockade in ALD, highlighting an area for development of host-targeted immunotherapeutic strategies in ALD, for which there are no current specific treatment options. This review aims at framing current knowledge on immune checkpoints and the possibility of their therapeutic utility in ALD-associated immune dysfunctions.

## Alcoholic liver disease

Alcoholic liver disease (ALD) is an escalating problem worldwide and is responsible for more than 3 million deaths annually, representing 5.9% of all deaths globally [1]. Alcohol-attributable deaths vary by continent and country, but Europe maintains the global record in terms of prevalence of alcohol consumption [2], with alcohol being responsible for 1 in 7 male deaths and 1 in 13 female deaths in the 15–64 year age group. In the UK alone, liver disease is the third biggest cause of premature mortality in the 18–64 year age group after ischemic heart disease and self-harm, with standardised mortality rates 4–5 times higher since the 1970s [3, 4]. Alcohol abuse also represents one of the strongest risk factors for the development of liver cancer [5], which is 16% more likely in those who drink heavily (> 5 units/day). Cancer Research UK estimates that liver cancer killed 4500 people in the UK in 2012, 3% of all cancer deaths [5]. Clearly, the potential societal impact of the development of effective therapeutic agents for ALD is far reaching.

There are currently no specific and efficacious therapeutic options for ALD patients, with abstinence being the cornerstone of treatment together with supportive care and liver transplantation currently indicated only for the most severe cases and available in very limited centres worldwide. This is compounded by a widespread reluctance to consider patients with advanced liver disease as candidates for transplantation [6].

Not surprisingly, abstinence is ineffective in completely reversing alcohol-related liver damage in patients who have been long-term excess alcohol abusers and who are at a more advanced and severe stage of disease. In this group, alcohol-related cirrhosis (ARC) is the most common form of established liver disease, which is accompanied by an increased risk of decompensation, organ failure and death. Moreover, high recidivism rates in abstinent patients can lead to repeated presentations of severe alcoholic hepatitis (SAH), the most florid form of ALD, a progressive inflammatory liver condition with a mortality rate of over 30% at 1 month post-hospitalization [7, 8] and approximately 60% during recidivism [9].

ALD is associated with multiple derangements in host immunity and it is now well-established that ARC induces a state of profound immunodeficiency, known as cirrhosis-associated immunodeficiency syndrome (CAIDS) [8, 10–12], which is accompanied by ongoing non-specific systemic inflammation, rendering ARC patients highly susceptible to overwhelming bacterial infections. This increases the risk of organ dysfunction including hepatic encephalopathy, renal failure and circulatory collapse, with no option for liver transplantation acutely [13, 14]. Alcohol abstinence does not fully resolve this, as once the liver is severely injured the deficiency in patients’ immunity remains and development of infection significantly compromises their survival chances [15].

Indeed, bacterial infections, sepsis and associated endotoxemia are diagnosed in 25–47% of hospitalized patients with cirrhosis and represent the most important triggers for acute decompensation and progression to multi-organ failure and septic shock, with short-term mortality of > 75% [11]. In SAH patients, the susceptibility to bacterial infection is further heightened and infection is observed in nearly 50% of cases in the short term with a high proportion of them ultimately dying of sepsis [16]. There is increasing evidence that changes in gut permeability, bacterial dysbiosis and translocation of bacteria from the ‘leaky’ gut into the systemic circulation in ALD is causatively linked to this increase in infections [17].

Current guidelines recommend early antibiotic therapy in these patients and suggest the use of corticosteroids to reduce the systemic inflammation associated with ALD [7, 8, 11, 16]. However, the intensive use of antibiotics has caused selection of multi-drug resistant bacteria, with some European centres reporting rates > 20% [18] and the STOPAH trial has clearly demonstrated lack of benefit with steroid treatment, both short term and long term [19]. Moreover, steroid treatment per se is associated with a further increased susceptibility to infection and sepsis [20].

It is critical to understand why ALD patients have impaired pathogen defence to develop targeted treatments to restore functional immunity. We have previously demonstrated that ALD patients harbour dramatic dysfunctions in their antibacterial defences, affecting both innate and adaptive immune cells (including neutrophils, monocytes, NK cells, T cells and Tregs) and also innate-like subsets (such as NKT cells and MAIT cells), and their responses to bacterial challenge are suboptimal and insufficient [21, 22] but reversible. Together, our findings have opened new avenues of research into the immunopathogenesis of ALD and have identified novel potential immunotherapeutic targets for the treatment of this complex disease, in particular the negative immune checkpoint receptors PD-1 and TIM-3 [23, 24]. We believe that host-directed immunomodulatory therapies aimed at restoring the dysfunctional immunity are a promising approach for the treatment of ALD. In this review, we aim to frame current knowledge on immune checkpoints and the possibility to use checkpoint blockade therapeutically in the context of ALD-associated immune dysfunctions.

## Immune responses are implicitly self-limiting

**Fig. 1:**
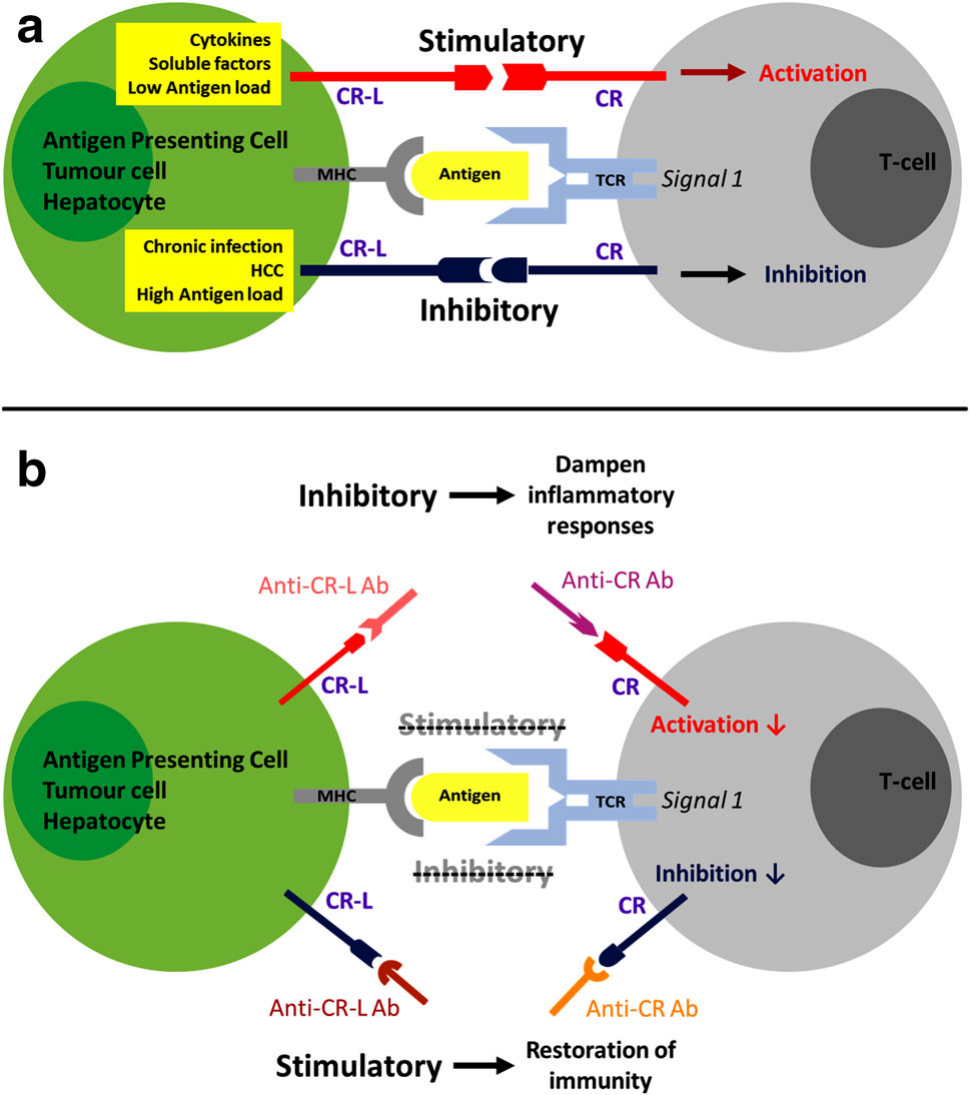
Immune regulation by checkpoint receptors (CR) and their ligands (CR–L), and effects of immune checkpoint blockade with neutralizing antibodies. Checkpoint receptors modulate the breadth, magnitude and spread of the immune response by balancing stimulatory and inhibitory signals delivered to immune cells by antigen-presenting cells or target cells (**a**). Blockade of immune checkpoint receptors or their ligands with neutralizing antibodies (Anti-CR Ab and Anti-CR–L Ab) can dampen inflammatory responses and restore dysfunctional immunity (**b**)

**Fig. 2:**
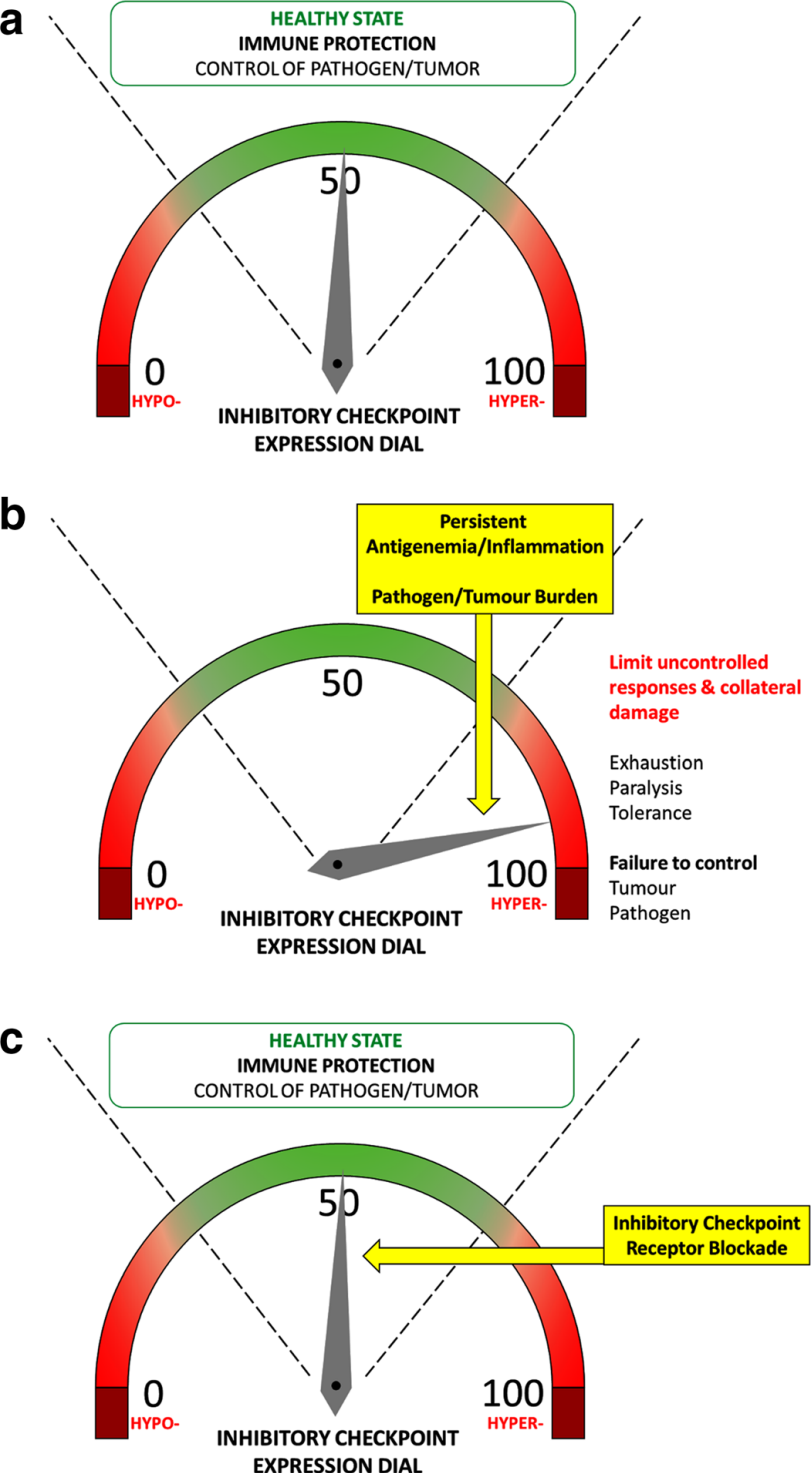
Increased expression of inhibitory checkpoint receptors suppresses homeostatic immunity and checkpoint blockade restores a healthy state. The homeostatic balance between anti-pathogen immunity and host-induced immunopathology is maintained in physiological conditions; this maintains a healthy immune state (**a**). Upon chronic stimulation, inhibitory checkpoints are hyper-expressed, limiting uncontrolled responses and immune-mediated damage but simultaneously suppressing efficient anti-pathogen responses (**b**). Blockade of inhibitory checkpoints can suppress these hyper-inhibitory signals, restoring a healthy immune state (**c**)

The immune system has a multitude of implicit mechanisms of self-regulation. Physiologically, these mechanisms promote immune tolerance and control unwanted and excessive injurious immune responses. This maintains immunological homeostasis, preventing immunopathology and limiting excessive inflammation and immune-mediated damage. A major arm of this multifaceted immunoregulatory network are the checkpoint receptors, a complex array of membrane receptors and their ligands that act as immune modulators, suppressing or activating key signal transduction pathways and modulating effector cell functions. By doing so, they fine-tune the magnitude, spread and breadth of the immune response, containing it and making it effective (Figs. 1a, 2a). Some of the main negative immune checkpoints that are currently being investigated in cancer, chronic viral infections and sepsis include the PD-1/PD-L1/PD-L2 pathway, the TIM-3/Galectin-9 pathway, CTLA-4 and LAG-3. However, there is an ever-increasing list of receptors and currently more than 20 endogenous immunoregulatory pathways have been identified and at least partly characterised [25].

Upon acute cell activation, these receptors appear on the cell surface concurrently with the acquisition of a very active functional profile (including secretion of multiple cytokines) [26] and are subsequently downregulated during the immune contraction phase, when the acute insults are resolved, tissue repair and wound healing mechanisms become predominant and immunological memory is consolidated. Instead, during chronic inflammation, chronic infections, cancer or sepsis, characterised by high levels of antigen and proinflammatory cytokines, multiple immunosuppressive checkpoint receptors are persistently hyper-expressed on the cell surface and are continuously activated, chronically suppressing immune cell functions. This phenomenon, called immune “exhaustion”, is characterised by a sequential loss of immune activities, including T-cell proliferation, secretion of cytokines and cytotoxic markers, and priming of pro-apoptotic pathways, causing a progressive immune shut-down [26, 27]. Furthermore, other immunocytes including B cells and NK cells are subjected to similar exhaustion processes, thereby extending the depth of immune suppression to humoral and innate immune responses [28–32]. The patients’ overly active immune system thus contains immunopathology and preserves the structural and functional integrity of tissues and organs but becomes unable to mount strong, effective and coordinated anti-pathogen responses (Fig. 2b). This favours susceptibility to infection, especially with opportunistic pathogens, similar to what we observe in septic patients and in ARC/SAH patients [10, 11, 33–35]. Immune functions remain persistently deranged for years after the resolution of the first septic episode in septic survivors and even after years of alcohol abstinence in ARC/SAH patients [15]. This is directly linked to persistently high expression of negative immune checkpoints [34].

Different immune checkpoints display different anatomical and temporal patterns of expression. The kinetics of immune checkpoint expression are highly regulated and coordinated and the dynamic interplay between stimulatory receptors (such as CD28, CD80 and CD86) and inhibitory checkpoints (e.g., PD-1, PD-L1, TIM-3) during cellular activation defines the evolution and fate of the existing immune response.

### The PD-1 and TIM-3 pathways

The PD-1 pathway belongs to the CD28/B7 family of T-cell co-receptors. PD-1/CD279 is probably the most studied checkpoint receptor in the field of T-cell exhaustion. This receptor was first identified in apoptotic T-cell lines (hence the name “programmed death 1”) but was soon characterised as a negative immunoregulator [36–39]. PD-1 has two known ligands, namely PD-L1/B7-H1/CD274 and PD-L2/B7-DC/CD273 [40–43]. PD-L1 is ubiquitously expressed at low levels and is strongly induced by proinflammatory signals [44, 45], while PD-L2 displays a more restricted expression profile [43, 46, 47]. Upon engagement, PD-1 sequesters intracellular factors involved in the TCR signalling, stopping T-cell activation [40–43]. PD-1/PD-L1 signalling appears to be bidirectional: PD-L1-expressing cancer cells possibly receive anti-apoptotic signals upon interaction with PD-1-expressing T cells [48, 49], but it is not known if this happens also in the context of T-cell interactions with antigen-presenting cells (APCs). PD-L1 also binds CD80, triggering inhibitory signals within PD-L1-expressing cells [50–53]. Amongst T cells, PD-1 is mostly expressed on primed T cells and is strongly upregulated upon TCR-mediated antigen-specific T-cell activation in peripheral tissues. Therefore, the PD-1 pathway is believed to play a role in the establishment and maintenance of peripheral tolerance [54]. It has been demonstrated that the PD-1 pathway can modulate immune cells other than T cells. The effect of PD-1 engagement on causing B cell exhaustion, for instance, is well-described [28, 29] and PD-1 expression on NK cells has also been linked to NK-cell functional suppression [31, 32]. In a study investigating immune exhaustion in HIV patients, contact with bacterial products induced monocyte expression of PD-1 and these monocytes secreted suppressive IL-10 upon PD-1 engagement. Furthermore, T-cell exhaustion in these patients could be reversed by blocking either PD-1 or IL-10 receptor [55]. Monocyte activation by bacterial endotoxin was also shown to physiologically cause increased secretion of suppressive IL-10 and upregulation of PD-1 and TIM-3 on T cells, and simultaneous blockade of TLR4 and CD14 abolished IL-10 secretion and inhibited T-cell checkpoint upregulation [21]. These findings link the PD-1 pathway to the regulation of antibacterial immunity, which is key in ALD patients.

TIM-3/CD366 was first described as a marker of activated IFNγ-producing T cells [56]. TIM-3 binds to Galectin-9 causing suppression of cytokine production, cell cycle arrest and even cell death [57, 58]. TIM-3 is widely expressed on several tissues and is promptly upregulated on T cells in response to both TCR-dependent and TCR-independent stimulation by common-gamma-chain cytokines (IL-2/7/15/21) [58–60]. Galectin-9 is widely expressed and is further induced by proinflammatory cytokines [61]. TIM-3 is important for T-cell exhaustion both in chronic viral infections [62–64] and in cancer [65–67]. TIM-3 is often co-expressed with PD-1 on severely exhausted T cells [25], where both receptors act synergistically to suppress immune functions [68, 69] and it has been demonstrated that blockade of TIM-3 partially restores these impaired T-cell functions [58, 66, 70]. However, there are still unanswered questions regarding paradoxical effects of the TIM-3 pathway observed in bacterial infection, for instance in tuberculosis where activation of this pathway seems to favour immune activation and disease control [71–73] but also suppression of T-cell functions and disease persistence [74]. Patients with SAH compared to healthy individuals display increased plasma Galectin-9 and higher TIM-3 expression on several immune subsets, together with production of suppressive IL-10 and reduced antibacterial functions, suggesting activation of the TIM-3 pathway in these patients [21].

### CTLA-4 and LAG-3

CTLA-4/CD152 is another member of the CD28/B7 family of T-cell co-receptors and is the first negative immune checkpoint studied in depth. CTLA-4 binds to CD80 and CD86 with approximately 20 times greater affinity than CD28 [40, 75], competing for CD80/CD86 binding and lowering the probability of T-cell costimulation by preventing activating interactions with APCs. Second, upon engagement the cytoplasmic tail of CTLA-4 sequesters factors involved in the TCR signalling, shutting down TCR-mediated T-cell activation [54, 76, 77]. Furthermore, CTLA-4 binding with CD80/CD86 can induce transendocytosis, effectively removing B7 molecules from the surface of APCs [78]. CTLA-4 can act bidirectionally, inducing the production of immunosuppressive IDO by APCs, which metabolically inhibits bystander T-cell functions [51]. CTLA-4 can also bind to another B7 family member called B7-H2, which is the only known ligand for the activatory receptor ICOS, possibly preventing ICOS-mediated T-cell costimulation [79]. Amongst T cells, CTLA-4 expression is stronger in naïve T cells and Tregs, and therefore it is believed that CTLA-4 in comparison to PD-1 may be more relevant in the initial phases of immune activation, preventing immune priming and the establishment/maintenance of central tolerance [54].

LAG-3/CD223 is a molecular homolog of CD4 [80], first described as a regulator of Treg activity [81]. LAG-3 binds uniquely to MHC-II molecules, which are upregulated during inflammation. The exact mechanisms of action of LAG-3 are still unclear. LAG-3 is strongly expressed on anergic and on exhausted T cells, often in strong association with PD-1 [82, 83]. Neutralizing antibodies against LAG-3 can only partially reverse anergy and rescue immune dysfunction [54, 84], but combined anti-LAG-3/PD-1 approaches have demonstrated stronger immune restoration [85], suggesting that LAG-3 inhibitory activity alone may be gentler than other inhibitory checkpoints.

## Immune checkpoints and checkpoint blockade in diseases

### Viral infections

Many pathogens have developed strategies to exploit immune checkpoint regulation as a way to facilitate immune escape/masking. For instance, viruses such as HIV, HCV and HBV, which establish chronic infections in humans, have evolved the ability to manipulate the PD-1 pathway to favour viral persistence [86–92]. In patients with chronic HBeAg + HBV infection, for example, we previously found dramatic T-cell dysfunctions associated with upregulation of PD-1 on virus-specific T cells [93]. PD-1 expression correlated directly with viremia and decreased progressively during antiviral treatment. In these patients, T cells displayed a skewed cytokine production with lack of antiviral IFNγ and predominant suppressive IL-10. During antiviral treatment, HBeAg + patients who achieved HBeAg seroconversion, which requires the presence of immunologically active T cells, appeared to be those with a more prominent loss of T-cell PD-1, suggesting immune reactivation, and the decreased PD-1 expression was accompanied by normalisation of cytokine production and IFNγ/IL-10 ratio. Higher expression of PD-1 on HBV-specific T cells has also been linked to failure to spontaneously eradicate the virus during acute infections, determining an immune milieu favourable to viral persistence [90, 94], while increased expression of PD-1 on B cells has been linked to B cell functional suppression in HIV infection [28, 29]. Blockade of the PD-1 pathway has been suggested as a possible host-targeted strategy to reactivate antiviral immunity and immune-mediated viral control in HBV, HIV or HCV infections (Figs. 1b, 2c) [86–92].

### Cancer

Blockade of negative checkpoints first received FDA approval in the context of anticancer treatments [54]. The tumor microenvironment expresses high levels of negative checkpoints and their ligands, which translates into a strong local suppression of anticancer responses. For instance, cancer cells of different origins express high levels of PD-L1 and IDO [95–100], either as a direct effect of cancer-related intracellular pathways [101, 102] or as a result of IFNγ stimulation by infiltrating immune cells [103–107]. Therefore, they can suppress tumor-infiltrating cancer-specific T cells by PD-1 engagement and by depleting the local milieu of essential tryptophan metabolites. Additionally, tumor-infiltrating lymphocytes have high expression of multiple checkpoint markers, with increasing numbers of receptors correlated to the severity of immune impairment [26]. Lastly, PD-1 is not only expressed on tumor-infiltrating T cells, but also on NK and B cells [54], suggesting a farther-reaching effect for a broader immune modulation.

In vitro checkpoint receptor blockade has demonstrated efficacy at rescuing exhausted tumor-specific T cells, favouring increased breadth and magnitude of effector functions and T-cell survival (Figs. 1b, 2c) [108] and an increasing number of clinical trials for new anticancer treatments based on immune checkpoint blockade are showing objective therapeutic responses in several patients, limiting disease progression and in some cases arresting or even reverting tumor growth. The most studied pathway in this context has been the B7 pathway [54, 109–112].

In pre-clinical studies, anti-CTLA-4 treatment showed successful reactivation of pre-existent anti-tumor immunity, alone or in combination with other immunomodulatory agents (such as GM-CSF) [54]. In clinical trials, treatment of melanoma with the FDA-approved anti-CTLA-4 antibody ipilimumab improved clinical outcome and survival both short term and long term, with relatively contained immune-related adverse events [113–116].

In a number of pre-clinical studies, PD-1/PD-L1 expression in the tumor microenvironment has been linked to immune dysfunction and PD-1/PD-L1 blockade has achieved immune rescue [54]. Clinically, anti-PD-1 and anti-PD-L1 have been tested in several cancers displaying good outcomes and a relatively good safety profile [111, 112]. The anti-PD-1 antibodies nivolumab and pembrolizumab have demonstrated better safety profiles and far greater success rates in comparison to current standard treatments (such as sorafenib to treat advanced hepatocellular carcinoma, HCC) [110] and also in comparison to anti-CTLA-4 strategies [54] in several clinical trials for different cancers [111, 117, 118]. In the ‘CheckMate 040’ trial for the use of nivolumab (humanised anti-PD-1 antibody) in advanced HCC 20% of patients had an objective response (OR), and up to 64% of them achieved disease control (DC), compared to < 1%OR and 43%DC, respectively, with sorafenib as standard treatment [110, 119]. PBMC and plasma/serum samples from the ‘CheckMate 040’ patients are being collected and stored in our laboratories and currently used to investigate a robust biomarker of treatment response in this cohort.

**Fig. 3:**
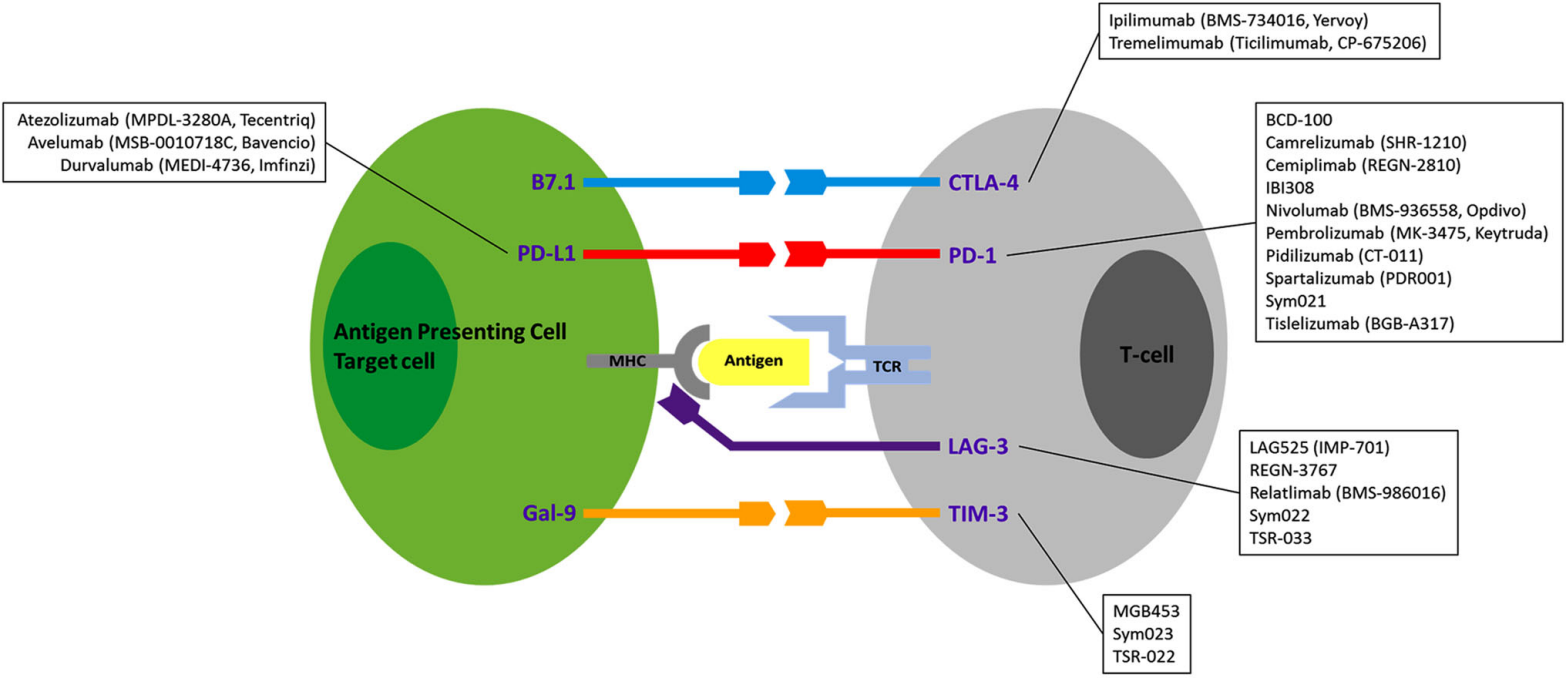
Immune checkpoints as therapeutic targets. Monoclonal antibodies currently in clinical development or tested in clinical trials against CTLA-4, PD-1, PD-L1, LAG-3 and TIM-3, as new anticancer agents, as these same immune checkpoint antibodies also represent the most promising therapeutic agents for future clinical trials in ALD

Inhibitory checkpoint antibodies are currently being tested alone or in combination with other neutralizing antibodies in cancer to achieve a stronger immune reconstitution by blocking several checkpoint receptors at once [54]. Inhibitory checkpoint antibodies currently in clinical development or tested in clinical trials as new anticancer agents are illustrated in Fig. 3 [120–124].

### Sepsis and septic shock

According to the Third International Consensus Definitions for Sepsis and Septic Shock (Sepsis-3), sepsis is a “life-threatening organ dysfunction caused by a dysregulated host response to infection (…) that arises when the body’s response to an infection injures its own tissues and organs” and septic shock is a “subset of sepsis in which particularly profound circulatory, cellular, and metabolic abnormalities are associated with a greater risk of mortality than with sepsis alone” [35]. The original biphasic description of sepsis and septic shock included a first phase during which an overwhelming systemic inflammatory response would develop upon pathogen detection (systemic inflammatory response syndrome, SIRS), characterised by uncontrolled immune activation, cytokine storm and the occurrence of immune-mediated tissue and organ damage, with potentially lethal consequences. A second phase would then ensue during which the hyper-active SIRS response would subside and compensatory anti-inflammatory mechanisms would complete pathogen clearance and tissue repair (compensatory anti-inflammatory response syndrome, CARS) [125–127], characterised by the induction of physiological mechanisms of immune shut-down and the activation of the healing response, with the acquisition of a strong endogenous immunosuppressed state. The current description of sepsis, however, has changed. It is now clear that SIRS and CARS are not two distinct sequential entities and the immune derangement that occurs during sepsis represents the contrasting combination of concurrent immune activation and immune exhaustion, where a state of persistent immunosuppression arises in parallel with the initial immune activation and drives the underlying immune dysfunction long term [128–132]. Indeed, septic patients who survive the initial inflammatory phase of sepsis present with an increased susceptibility to secondary opportunistic infections long term, indicating that immunosuppression and immune impairments are maintained over time [133, 134]. Immunosuppression rather than hyper-immunity drives the response to sepsis, as also supported by the evidence that clinical trials focussed on reducing hyper-immunity/SIRS have provided conflicting and disappointing results [135–138].

Negative immune checkpoints (including PD-1, PD-L1, TIM-3, CTLA-4, LAG-3 and others) play a causal role in this persistent immunosuppression [139–143]. Their expression on both innate and adaptive immune cells is greatly increased in septic patients, correlating with loss of immune functions (including innate antibacterial activities from monocytes, macrophages or neutrophils and T-cell production of cytokines and cytotoxic factors), immune cell apoptosis, reduced pathogen clearance and increased patient mortality [34, 141, 143–146]. Most of these immune dysfunctions can be at least partially restored by blocking checkpoint pathways (Figs. 1b, 2c) [34]. This strategy is currently being investigated in several pre-clinical and clinical ex vivo studies with promising results in septic patients, suggesting that host-targeted immunotherapy may rescue suppressed antimicrobial immunity, reduce susceptibility to infection and improve patient survival [34]. The first clinical trial to determine the safety profile and efficacy of treatment with the anti-PD-1 antibody nivolumab in patients with severe sepsis or septic shock has been completed (NCT02960854), and results of this trial are eagerly awaited.

## Immune checkpoints and checkpoint blockade in ALD

Many features of sepsis and septic shock resemble those observed in ALD patients who acquire bacterial infection. This is particularly pertinent in the context of severe ALD, including decompensated cirrhosis, alcohol-related liver failure, alcohol-related acute-on-chronic liver failure (ACLF) and SAH. Furthermore, in abstinent patients immune defects persist over a long term, a common feature with sepsis survivors. Hence, there may be a strong parallelism between mechanisms of immune dysfunction in sepsis and those at play in ALD. As discussed, several studies have investigated the contribution of negative immune checkpoints to the immunopathophysiology of sepsis and several pre-clinical and clinical studies are defining the parameters of immune checkpoint blockade as a therapeutic strategy in these patients. However, no such clinical investigations exist in the context of ALD, highlighting a large gap in the possibility to develop new host-targeted strategies for ALD and its complications, for which there are no current specific treatment options.

In a 2015 study, we performed an in-depth ex vivo immunological characterisation of antibacterial responses in ARC and SAH patients and we were the first group to show that immune dysfunctions observed in SAH patients relate directly to increased expression of PD-1 and TIM-3 on several immune subsets [21]. Immune alterations were directly correlated with severity of disease, and gut-derived bacterial products were driving these immune dysfunctions, therefore highlighting a parallel with bacterial sepsis.

First, we observed that neutrophils had reduced bacterial phagocytosis and increased non-specific ROS production, which may cause bystander tissue damage in the inflamed liver. Furthermore, when challenged with bacterial cells and antigens, neutrophils were unable to mount an oxidative response, indicating a defect in antibacterial innate functions.

Upon bacterial stimulation, we observed that antibacterial responses were predominantly immunosuppressive in SAH patients, with less IFNγ-producing and more IL-10-producing cells compared to healthy individuals. This immune imbalance was directly correlated with increased expression of PD-1 and TIM-3 on both CD4 and CD8 T cells and, in addition, plasma levels of TIM-3 ligand Galectin-9 were increased in SAH patients. PD-1 and TIM-3 were also increased on NK and NKT cells. It remains unclear whether PD-1 and TIM-3 or checkpoint receptors in general have a role in modulating humoral responses in ALD.

When we investigated the causes of PD-1/TIM-3 hyper-expression we found that both ethanol alone and stimulation with bacterial endotoxin dose-dependently upregulated both immune checkpoints on CD4, CD8 T cells and Tregs. The effects of ethanol and endotoxin were additive, and skewed cytokine production preferentially towards IL-10 production.

Since monocytes are amongst the most endotoxin-responsive immune cells, we performed blocking experiments directed at suppressing TLR4/CD14 signalling, and we observed that combined blockade of both endotoxin receptors abolished IL-10 and TNFα monocyte production upon bacterial challenge and above all completely prevented PD-1/TIM-3 upregulation on CD4 and CD8 T cells.

Most importantly, blockade of PD-1 and TIM-3 in endotoxin-stimulated PBMCs restored IFNγ and reduced IL-10 production in SAH patients, re-establishing an appropriate IFNγ/IL-10 balance. Furthermore, checkpoint blockade increased the phagocytic and oxidative neutrophil response to bacterial challenge, suggesting that immune dysfunctions in ALD patients are not permanent but reversible and that immune checkpoint blockade may be useful to restore defective antibacterial immunity in ALD patients, especially in SAH patients.

The involvement of the PD-1 pathway in causing skewed IL-10 production and pathogenic immunosuppression is already known in patients with non-alcoholic sepsis, where endotoxin-driven IL-10 production and IFNγ suppression can be reversed by therapeutic PD-1/PD-L1 blockade, with improved bacterial clearance and reduced patient mortality [147, 148]. Endotoxin levels in the liver are higher compared to the periphery [149] and liver inflammation correlates with intrahepatic expression of PD-1/PD-L1 [150]. Interestingly, in mouse models of sepsis blockade of the PD-1 pathway reduced liver inflammation and increased survival [148, 151].

In our study, immune reconstitution driven by in vitro PD-1/TIM-3 blockade was not accompanied by exacerbation of inflammatory markers (including IL-1β/6/8, TNFα and IP-10, which are linked to immunopathology in SAH) and did not increase spontaneous ROS production in neutrophils, suggesting that immune checkpoint blockade may be a safe therapeutic approach in ALD [21]. Anti-checkpoint antibodies currently used for therapeutic purposes in cancer and sepsis have good safety profiles, with low occurrence of severe adverse events, and it could be argued that good safety profiles may be maintained when these treatment strategies are extended to ALD.

## Risks of checkpoint receptor therapy, biomarkers and new developments

Immunotherapy with inhibitory checkpoint blockade raises the possibility of skewing immunity towards an injurious hyper-active response, with increased inflammation and loss of immune tolerance, resulting in immune-related adverse events (irAEs). Moreover, breaking tolerance in patients who already harbor severe immune dysfunction, such as advanced ALD patients, may further exacerbate this risk.

In our study we did not observe exacerbation of inflammatory markers following immune checkpoint blockade ex vivo, as described earlier [21]. No information is currently available on irAEs derived from immune checkpoint blockade in sepsis and septic shock, as the NCT02960854 clinical trial remains unpublished at the time of writing, but in the context of liver studies, the majority of irAEs reported in the ‘CheckMate 040’ clinical trial for the treatment of hepatocellular carcinoma with the anti-PD-1 antibody nivolumab were mild (mainly dermatological and gastrointestinal; 75% below grade 3), with low occurrence of severe irAEs (6%) and no treatment-related deaths [110]. Overall, the ‘CheckMate 040’ safety results are in line with most studies reviewing the occurrence of irAEs following immune checkpoint blockade [152, 153].

The development of irAEs clearly indicates that blocking inhibitory checkpoint pathways is effective at reactivating a dormant immune system. Conversely, a heightened state of immunity and inflammation can also have an effect on checkpoint blockade responses. In fact, it has been suggested that pre-existing autoimmune conditions may render some patients more susceptible to developing irAEs, although it is still unclear whether irAEs correlate with successful treatment response [152, 153]. On the other hand, some patients are refractory to treatments using checkpoint blocking antibodies, which has been linked, for example, to defective antigen presentation to effector T cells, greater T-cell exhaustion and T-cell expression of a broader array of inhibitory checkpoints or enhanced proinflammatory signalling via interferon responsive elements [154–156].

Overall, accurate patient selection is a key. Objective responses to immune checkpoint-based treatments are expected to be at least partly dependent on the presence of pre-existing checkpoint-driven immune dysfunctions and on the expression of high levels of checkpoint receptors in the first place. Meta-analyses of checkpoint blockade treatment in cancer have clearly shown this to be the case and that, for instance, higher expression levels of PD-L1 by the tumor microenvironment are associated with better response to anti-PD-1 treatment and lower occurrence of adverse events in a variety of cancers [157, 158], although agreement on this topic is not absolute [159, 160]. Regardless, monitoring levels of immune checkpoint expression prior to initiating treatment may provide additional indications for a more accurate selection of patients who are more likely to have a response without developing immunopathology [157, 158].

A further complication resides in the fact that immune checkpoint receptors and their ligands can also exist in soluble form [161]. Proposed mechanisms involved in the generation of these soluble forms include alternative mRNA splicing or protease-mediated shedding and it is still unclear which mechanism prevails for which specific immune checkpoint [161]. Similarly, it is unclear how soluble immune checkpoints act, whether they are partial or full agonists for their membrane-bound ligands, or rather simple antagonist decoys for each other or for their membrane-bound counterparts [161]. The presence of soluble checkpoints has been demonstrated in several disorders, often in correlation with disease severity and response to treatment, but current studies are far from providing conclusive results [162–169]. Soluble checkpoint receptors and ligands may be ideal candidate diagnostic and prognostic biomarkers and it will be interesting to investigate if and how they affect response to checkpoint blockade treatment and in particular if and how they can be a priori predictors of positive outcome [170].

## Perspectives and conclusions

The immune checkpoint system is a very complex and exquisitely fine-tuned endogenous network of immune regulation. The balance between protective immunity and immune tolerance on one side and immunopathology and autoimmunity on the other relies on a dynamic homeostatic equilibrium between co-stimulatory signals and a comparatively larger number of inhibitory pathways, which can be easily skewed causing strong and persistent immunosuppression during chronic inflammatory diseases. Chronic infection, cancer, sepsis and ALD present with partly overlapping immune profiles, characterised by hyper-expression of inhibitory checkpoint pathways on several subsets of immune cells and consequent innate and adaptive immune defects, which depending on the disease will favour immune escape, immune masking or inability to contend with secondary infections. Novel host-targeted therapies based on blocking negative immune checkpoints are efficacious and have good safety profiles in cancer treatment and are being tested to resolve persistent immunosuppression in septic patients. We have been the first to describe a role for PD-1 and TIM-3 in the immunopathogenesis of ALD and we believe that these and other membrane and soluble immune checkpoints will represent novel potential prognostic markers and safe therapeutic targets for the treatment of ALD.

## Abbreviations

ARC: Alcohol-related cirrhosis
APC: Antigen-presenting cell
CD: Cluster of differentiation
CTLA-4: Cytotoxic T-lymphocyte associated protein 4
IFN: Interferon
IL: Interleukin
LAG-3: Lymphocyte-activation gene 3
MHC: Major histocompatibility complex
MAIT: Mucosa-associated invariant T cells
NK: Natural killer cells
NKT: Natural killer T cells
PBMC: Peripheral blood mononuclear cells
PD-1: Programmed death 1
PD-L1/2: Programmed death ligands 1 and 2
ROS: Reactive oxygen species
SAH: Severe alcoholic hepatitis
TIM-3: T cell immunoglobulin and mucin domain 3
HAVCR2: Hepatitis A virus cellular receptor 2
TNF: Tumor necrosis factor
